# Treatise on the Dental Art, Founded on Actual Experience

**Published:** 1843-07

**Authors:** 


Art. III.—*Treatise on the Dental Art, founded on Actual Experience.*—Illustrated by two hundred and forty-one figures in Lithography, and fifty-four Wood-cuts. By F. Maury, Dentist of the Royal Polytechnic School. Translated from the French, with notes and additions, by J. B. Savier, Doctor of Dental Surgery. Philadelphia: Lea and Blanchard, 1842; p. 324.

We are neither a dentist nor the son of a dentist (and for the former at least we devoutly return thanks); nevertheless we take an interest in whatever relates to this branch of the healing art. It gives us pleasure, therefore, to notice the book named above. It is of a highly practical character, and from the value accorded to it abroad, will prove a welcome addition to the Dental literature of this country. If we were to find fault with it, we should say that it was too concise or too meagre in that part devoted to the pathology of the mouth and its appendages. But we say this with hesitation, and certainly would not insist on it. C.

